# Supplementary material for: Identification and functional analysis of non-coding regulatory small RNA FenSr3 in Bacillus amyloliquefaciens LPB-18
Source: PeerJ. 2023 May 15;11:e15236. doi: 10.7717/peerj.15236 (PMC10194069; doi:10.7717/peerj.15236)
Supplement: Supplemental Information 4 [file peerj-11-15236-s004.zip › KO/CK-vs-T1_map/map00905.html]

KEGG PATHWAY: Brassinosteroid biosynthesis - Reference pathway


|  |  |
| --- | --- |
| **Brassinosteroid biosynthesis - Reference pathway** |  |

[
Pathway menu
| Organism menu
| Pathway entry
| Show description
| User data mapping
]

Brassinosteroids are a group of plant steroid hormones that regulate growth and development. More than fifty naturally occurring brassinosteroids have been identified in a wide range of plant species. The most abundant and widely occurring brassinosteroids are C28 steroids, and among them brassinolide (BL) is the most biologically active. Plants have multiple pathways for biosynthesis of BL, which are derived from the steroid biosynthetic pathway. Two pathways from campestanol to castasterone (CS), C6 oxidation and the late-C6 oxidation pathways, operate in many plants. Another branching pathway, the early-C22 oxidation pathway, was demonstrated using a brassinosteroid-deficient mutant of Arabidopsis thaliana. Recently, a new shortcut pathway from campesterol to 6-deoxotyphasterol was demonstrated by a functional analysis of cytochrome P450 monooxygenases responsible for brassinosteroid biosynthesis. Thus, at least four pathways are involved in the biosynthesis of CS, and CS is further metabolized to BL by lactonization of the B ring.

|  |  |  |
| --- | --- | --- |
| Reference pathway | 184% 150% 122% 100% 82% 67% 55% | 图片下载 |
